# Prevalence of Screening for Food Insecurity, Housing Instability, Utility Needs, Transportation Needs, and Interpersonal Violence by US Physician Practices and Hospitals

**DOI:** 10.1001/jamanetworkopen.2019.11514

**Published:** 2019-09-18

**Authors:** Taressa K. Fraze, Amanda L. Brewster, Valerie A. Lewis, Laura B. Beidler, Genevra F. Murray, Carrie H. Colla

**Affiliations:** 1 The Dartmouth Institute for Health Policy and Clinical Practice, Geisel School of Medicine, Dartmouth College, Lebanon, New Hampshire; 2 School of Public Health, Division of Health Policy and Management, University of California, Berkeley; 3 Gilling School of Global Public Health, Department of Health Policy and Management, University of North Carolina at Chapel Hill

## Abstract

**Importance:**

Social needs, including food, housing, utilities, transportation, and experience with interpersonal violence, are linked to health outcomes. Identifying patients with unmet social needs is a necessary first step to addressing these needs, yet little is known about the prevalence of screening.

**Objective:**

To characterize screening for social needs by physician practices and hospitals.

**Design, Setting, and Participants:**

Cross-sectional survey analyses of responses by physician practices and hospitals to the 2017-2018 National Survey of Healthcare Organizations and Systems. Responses were collected from survey participants from June 16, 2017, to August 17, 2018.

**Exposures:**

Organizational characteristics, including participation in delivery and payment reform.

**Main Outcomes and Measures:**

Self-report of screening patients for food insecurity, housing instability, utility needs, transportation needs, and experience with interpersonal violence.

**Results:**

Among 4976 physician practices, 2333 responded, a response rate of 46.9%. Among hospitals, 757 of 1628 (46.5%) responded. After eliminating responses because of ineligibility, 2190 physician practices and 739 hospitals remained. Screening for all 5 social needs was reported by 24.4% (95% CI, 20.0%-28.7%) of hospitals and 15.6% (95% CI, 13.4%-17.9%) of practices, whereas 33.3% (95% CI, 30.5%-36.2%) of practices and 8.0% (95% CI, 5.8%-11.0%) of hospitals reported no screening. Screening for interpersonal violence was most common (practices: 56.4%; 95% CI, 53.3%-2 59.4%; hospitals: 75.0%; 95% CI, 70.1%-79.3%), and screening for utility needs was least common (practices: 23.1%; 95% CI, 20.6%-26.0%; hospitals: 35.5%; 95% CI, 30.0%-41.0%) among both hospitals and practices. Among practices, federally qualified health centers (yes: 29.7%; 95% CI, 21.5%-37.8% vs no: 9.4%; 95% CI, 7.2%-11.6%; *P* < .001), bundled payment participants (yes: 21.4%; 95% CI, 17.1%-25.8% vs no: 10.7%; 95% CI, 7.9%-13.4%; *P* < .001), primary care improvement models (yes: 19.6%; 95% CI, 16.5%-22.6% vs no: 9.6%; 95% CI, 6.0%-13.1%; *P* < .001), and Medicaid accountable care organizations (yes: 21.8%; 95% CI, 17.4%-26.2% vs no: 11.2%; 95% CI, 8.6%-13.7%; *P* < .001) had higher rates of screening for all needs. Practices in Medicaid expansion states (yes: 17.7%; 95% CI, 14.8%-20.7% vs no: 11.4%; 95% CI, 8.1%-14.6%; *P* = .007) and those with more Medicaid revenue (highest tertile: 17.1%; 95% CI, 11.4%-22.7% vs lowest tertile: 9.0%; 95% CI, 6.1%-11.8%; *P* = .02) were more likely to screen. Academic medical centers were more likely than other hospitals to screen (49.5%; 95% CI, 34.6%-64.4% vs 23.0%; 95% CI, 18.5%-27.5%; *P* < .001).

**Conclusions and Relevance:**

This study’s findings suggest that few US physician practices and hospitals screen patients for all 5 key social needs associated with health outcomes. Practices that serve disadvantaged patients report higher screening rates. The role of physicians and hospitals in meeting patients’ social needs is likely to increase as more take on accountability for cost under payment reform. Physicians and hospitals may need additional resources to screen for or address patients’ social needs.

## Key Points

**Question:**

What types of physician practices and hospitals self-report screening patients for food, housing, transportation, utilities, and interpersonal violence needs?

**Findings:**

In a cross-sectional study of US hospitals and physician practices, approximately 24% of hospitals and 16% of physician practices reported screening for food insecurity, housing instability, utility needs, transportation needs, and interpersonal violence. Federally qualified health centers and physician practices participating in bundled payments, primary care improvement models, and Medicaid accountable care organizations screened more than other hospitals, and academic medical centers screened more than other practices.

**Meaning:**

This study’s findings suggest that most US physician practices and hospitals do not report screening patients for key social needs, and it appears that practices serving more economically disadvantaged populations report screening at higher rates.

## Teaser

This cross-sectional study uses national survey data to assess the percentage of US hospitals and physician practices that screen patients for food insecurity, housing instability, utility needs, transportation needs, and interpersonal violence and to compare characteristics between screening and nonscreening entities.

## Introduction

High-quality, coordinated medical care alone cannot ensure that patients achieve optimal health outcomes.^1^ Up to 90% of health outcomes are a result of social, behavioral, and economic factors.^2^ The association of patients’ social needs, such as food insecurity, housing instability, utility needs, transportation needs, and experience with interpersonal violence, with health outcomes and costs is increasingly recognized by the medical community, and an increasing amount of evidence documents that physician- and hospital-led interventions addressing patients’ social needs can produce improved health outcomes and less costly medical care.^3,4,5,6^ With the goal of improving integration of social needs–informed care into medical care, the American Academy of Family Physicians, the National Association of Community Health Centers, and the American Academy of Pediatrics have launched campaigns to encourage physicians to screen patients for social needs.^7,8,9^

Historically, systematically identifying and addressing patients’ social needs has not been part of medical practice.^1^ Although physicians and hospitals may recognize the association of social needs with patient outcomes, they may be reluctant to assume responsibility for social needs given the complexity of addressing these needs coupled with increasing clinical demands.^10,11^ Despite these challenges, the movement toward value-based care has accelerated the focus of physicians and hospitals on population and whole-person health by incentivizing physicians and hospitals to address needs that may be outside of traditional clinical care yet associated with health spending.^3,12^ Furthermore, state and federal policymakers as well as private payers are designing programs aimed at integrating social services into clinical care. For example, the Centers for Medicare & Medicaid Services (CMS) created the Accountable Health Community model, and multiple states have recently established waivers that allow Medicaid dollars to pay for services that support patients’ social needs.^13,14,15^ At the same time, as part of their campaigns to encourage physicians and hospitals to screen patients for social needs, the American Academy of Family Physicians,^7^ National Association of Community Health Centers,^9^ and the American Academy of Pediatrics^8^ have each developed tools aimed at helping physicians and hospitals identify patients with social needs and then offer them support to avail themselves of community resources. Despite support by physician and hospital groups coupled with delivery reform from payers, little is known about the extent to which these initiatives have diffused into care delivery.

Timely, national data are needed to better understand the extent to which physicians and hospitals are embracing recommendations to screen patients for social needs. Previous research has largely focused on specific interventions or in-depth assessments of how some physicians and hospitals address social needs.^3,16,17^ Although many health care organization leaders have voiced their commitment to addressing social needs, no national data exist on how often and which types of physicians and hospitals screen patients for social needs. In this study, we used new, nationally representative survey data to assess the prevalence of screening among physician practices and hospitals for social needs, specifically housing instability, food insecurity, utility needs, transportation needs, and the experience with interpersonal violence. We examined how screening efforts vary by organizational characteristics and capabilities, and we identified major barriers to adopting care delivery innovations reported by physicians and hospitals. A better understanding of the current landscape of social needs screening and of the nature of organizations who screen will help stakeholders determine physician and hospital readiness to engage, implement, and scale programs aimed at addressing patients’ social needs.

## Methods

This study followed the Strengthening the Reporting of Observational Studies in Epidemiology (STROBE) reporting guideline.^18^ We used novel data from the National Survey of Healthcare Organizations and Systems (NSHOS),^19^ supplemented by additional data from the OneKey database (IQVIA Inc),^20^ the American Hospital Association’s (AHA) Annual Survey,^21^ and the US Census^22^ to characterize US physician practices and hospitals that screen patients for social needs. The NSHOS, which actively accepted survey responses from June 16, 2017, to August 17, 2018, is a suite of nationally representative surveys, including a survey of physician practices with 3 or more primary care physicians and a survey of nonspecialty, acute care hospitals. The NSHOS collected data on the structure, ownership, leadership, and care delivery capabilities of these organizations.^19^ This study was approved Dartmouth College’s institutional review board, which waived informed consent for NSHOS because risk to respondents was low and the survey asked them to respond in their professional capacity.

### Sampling and Data Collection

The NSHOS used the OneKey database,^20^ produced by IQVIA, to identify and sample physician practices and hospitals. OneKey relies on proprietary data collection efforts, the American Medical Association’s Physician Masterfile, and publicly available sources. OneKey provides information on the relationships among physicians, practices, hospitals, and health care systems. The NSHOS used a stratified-cluster sampling design that drew samples of physician practices and hospitals with different ownership and composition structures, including samples of both system-owned and independent physician practices and hospitals. The NSHOS includes a single response from each of 2190 physician practices and 739 acute care or critical access hospitals.

In the physician practice survey, the NSHOS surveyed practices with at least 3 primary care physicians, with primary care defined as family medicine, geriatrics, internal medicine, or preventive medicine specialties. Practices that also included specialty physicians were part of the sample. The target respondent was a medical director, physician, or practice manager. The NSHOS sampled 4976 physician practices and obtained a response from 2333 practices, for a response rate of 46.9%. We removed 143 responses because of ineligibility, which left 2190 responses to analyze.

In the NSHOS hospital survey, hospitals included short-term acute care and critical access hospitals; specialty hospitals were excluded. The NSHOS sampled 1628 hospitals and obtained a response from 757 hospitals, for a response rate of 46.5%. We removed 18 responses because of ineligibility, which left 739 responses to analyze. Target hospital respondents were leaders, such as the chief medical officer, chief executive officer, or other C-suite leaders.

### Main Outcome Measures

To assess screening for social needs among physician practices and hospitals, the NSHOS asked “Does your practice have a system in place to screen patients for food insecurity (yes/no), housing instability (yes/no), utility needs (yes/no), transportation needs (yes/no), or interpersonal violence (yes/no)?” Hospitals were asked a similar question. We selected these 5 domains because these are part of the CMS’s Accountable Health Communities model. We characterized physician practices and hospitals that report screening patients for all 5 social needs.

Physician practices were characterized using data from the NSHOS, OneKey, and the US Census. Characteristics of practices included practice ownership, physician composition, type of practice, share of Medicaid revenue in the practice (by tertile), region, rural (those with ≤49 999 residents) or metropolitan area, and participation in payment and delivery reform initiatives, including bundled payments, primary care improvement models (eg, medical homes, comprehensive primary care), and accountable care organizations (ACOs).

We characterized hospitals using NSHOS, OneKey, the AHA Annual Survey, and US Census data.^19,20,21,22,23^ Measures from the AHA included hospital ownership, share of inpatient admissions that were covered by Medicaid, and participation in an ACO contract. The NSHOS measures included academic vs nonacademic hospital, critical access status, region, and revenue received from episode-based payments. We used US Census data to obtain the area-level share of residents whose income was below the federal poverty level, which we divided into tertiles, and metropolitan vs rural areas.

We compared barriers to care delivery innovations that physician practices and hospitals reported by screening status. Respondents reported whether potential barriers were not a barrier, were a minor barrier, or were a major barrier to their use of care-delivery innovations. Innovations were not specific to screening for social needs. Barriers focused on processes to identify or disseminate innovations and lack of time, financial resources, knowledge, or incentives.

### Statistical Analysis

We characterized physician practices and hospitals screening for all 5 social needs by using means and proportions with 95% CIs. We used χ^2^ tests to calculate significance. Significance was set at 2-sided *P* = .05. As a sensitivity analysis, we compared physician practices with hospitals that screened for food insecurity, housing instability, and utility needs; that screened for food insecurity, housing instability, utility needs, and transportation needs; and that did not screen for any needs. We excluded survey respondents with missing data for a given variable.

We used the OneKey database to weight responses from physician practices to all practices with 3 or more primary care physicians and from hospitals to all acute care, nonspecialty hospitals that met our sample frame definition. Data were weighted to adjust for nonresponse bias. The OneKey data allowed us to adjust responses based on practices’ and hospitals’ ownership, structure, and composition. Statistical calculations were performed using Stata Statistical Software, Release 13 (StataCorp).

## Results

**Figure 1.**
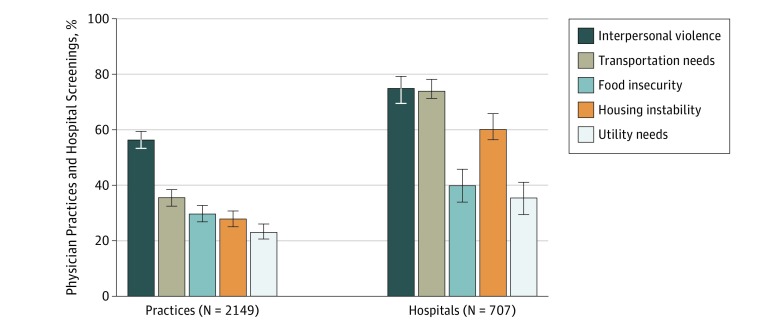
Percentage of Physician Practices and Hospitals That Screen Patients for Each of 5 Social Needs Number of survey respondents from the 2017-2018 National Survey of Healthcare Organizations and Systems are unweighted. Percentages represent weighted data; error bars, 95% CIs.

**Figure 2.**
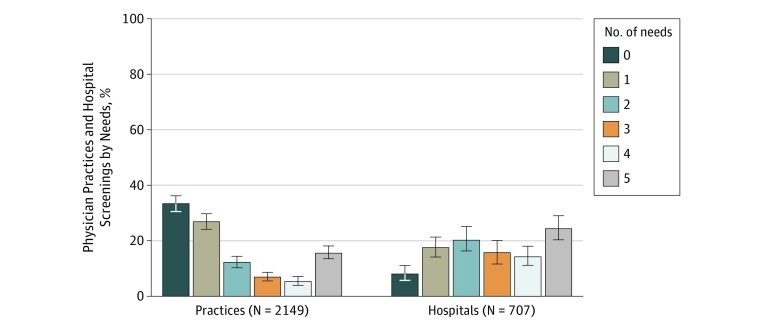
Percentage of Physician Practices and Hospitals That Screen Patients for Food Insecurity, Housing Instability, Utility Needs, Transportation Needs, and Experience With Interpersonal Violence, by Number of Needs Screened Number of survey respondents from the 2017-2018 National Survey of Healthcare Organizations and Systems are unweighted. Percentages represent weighted data; error bars, 95% CIs.

Among 4976 physician practices, 2333 responded, a response rate of 46.9%. Among hospitals, 757 of 1628 (46.5%) responded. After eliminating responses because of ineligibility, 2190 physician practices and 739 hospitals remained. Screening rates varied by social need: 56.4% (95% CI, 53.3%-59.4%) of physician practices reported screening for interpersonal violence, 35.4% (95% CI, 32.5%-38.4%) for transportation needs, 29.6% (95% CI, 26.8%-32.7%) for food insecurity, 27.8% (95% CI, 27.8%-25.0%) for housing instability, and 23.1% (95% CI, 20.6%-26.0%) for utility needs. For hospitals, 75.0% (95% CI, 70.1%-79.3%) reported screening for interpersonal violence, 74.0% (95% CI, 69.2%-78.2%) for transportation needs, 60.1% (95% CI, 54.2%-65.8%) for housing instability, 39.8% (95% CI, 34.2%-45.7%) for food insecurity, and 35.5% (95% CI, 30.0%-41.0%) for utility needs (Figure 1). Most physician practices and hospitals did not screen for all 5 needs; 15.6% (95% CI, 13.4%-17.9%) of physician practices and 24.4% (95% CI, 20.0%-28.7%) of hospitals reported screening for all 5 social needs, whereas 33.3% (95% CI, 30.5%-36.2%) of physician practices and 8.0% (95% CI, 5.8%-11.0%) of hospitals reported screening for no social needs (Figure 2). Much of the overall screening activity was driven by screening for interpersonal violence: when screening for a single need, 74.2% (95% CI, 68.0%-79.5%) of physician practices and 57.3% (95% CI, 45.8%-68.2%) of hospitals reported screening only for experience with interpersonal violence.

### Characteristics of Practices That Screen for Social Needs

Physician practices that serve more disadvantaged patients, including federally qualified health centers (yes: 29.7%; 95% CI, 21.5%-37.8% vs no: 9.4% ;95% CI, 7.2%-11.6%; *P* < .001) and those with more Medicaid revenue (9.0% for the lowest tertile; 95% CI, 6.1%-11.8% vs 17.1% for the highest tertile; 95% CI, 11.4%-22.7%; *P* = .02), were more likely to screen for all 5 social needs (Table 1). Practices in a Medicaid ACO contract (yes: 21.8%; 95% CI, 17.4%-26.2%; vs no: 11.2%; 95% CI, 8.6%-13.7%; *P* < .001) and Medicaid expansion states (yes: 17.7%; 95% CI, 14.8%-20.7% vs no: 11.4%; 95% CI, 8.1%-14.6%; *P* = .007) were also more likely to screen for all 5 social needs. Physician practices that participated in bundled payments (yes: 21.4%; 95% CI, 17.1%-25.8% vs no: 10.7%; 95% CI, 7.9%-13.4%; *P* < .001); primary care improvement models, such as medical homes (yes: 19.6%; 95% CI, 16.5%-22.6% vs no: 9.6%; 95% CI, 6.0%-13.1%; *P* < .001); or a commercial ACO contract (yes: 18.4%; 95% CI, 15.0%-21.8% vs no: 12.4%; 95% CI, 9.0%-15.7%; *P* = .02) had higher rates of screening for all needs. Practices in the West had the highest screening rates (19.7%; 95% CI, 13.9%-25.4%) followed by the South (16.1%; 95% CI, 11.9%-20.2%), the Northeast (16.0%; 95% CI, 11.0%-21.0), and the Midwest (10.8%; 95% CI, 7.9%-13.6%) (*P* = .05). We observed no significant differences in screening for all needs by practice size, inclusion of specialists, rural status, or ownership.

**Table 1.** Characteristics of Physician Practices That Screen for Food Insecurity, Housing Instability, Utility Needs, Transportation Needs, and Experience With Interpersonal Violence

Characteristic	Screen for All 5 Needs		
	No.^a^	Weighted % (95% CI)	*P* Value
Overall	346	15.6 (13.4-17.9)	
Practice ownership^b^			
Independent	87	14.8 (10.4-19.1)	.57
System	259	16.3 (13.9-18.7)	
Academic medical center affiliation^c^^,^^d^			
Yes	129	17.9 (14.0-21.9)	.20
No	217	14.8 (12.1-17.6)	
Practice type^c^			
Multispecialty	167	16.0 (12.5-19.6)	.76
Primary care only	179	15.3 (12.4-18.2)	
Federally qualified health center^c^			
Yes	83	29.7 (21.5-37.8)	<.001
No	148	9.4 (7.2-11.6)	
Medicaid expansion state^e^			
Yes	262	17.7 (14.8-20.7)	.007
No	84	11.4 (8.1-14.6)	
Medicaid revenue^c^			
<5%	69	9.0 (6.1-11.8)	.02
5%-20%	86	14.6 (10.7-18.5)	
>20%	87	17.1 (11.4-22.7)	
Region^f^			
South	90	16.1 (11.9-20.2)	.05
Midwest	80	10.8 (7.9-13.6)	
West	96	19.7 (13.9-25.4)	
Northeast	80	16.0 (11.0-21.0)	
Area^g^			
Rural	45	14.4 (9.1-19.8)	.64
Metropolitan	300	15.9 (13.3-18.4)	
Bundled payments^c^^,^^h^			
Yes	156	21.4 (17.1-25.8)	<.001
No	130	10.7 (7.9-13.4)	
Primary care improvement model^c^			
Yes	261	19.6 (16.5-22.6)	<.001
No	65	9.6 (6.0-13.1)	
Medicare ACO^c^^,^^h^			
Yes	201	15.8 (12.9-18.8)	.53
No	102	14.3 (10.3-18.2)	
Medicaid ACO^c^			
Yes	176	21.8 (17.4-26.2)	<.001
No	132	11.2 (8.6-13.7)	
Commercial ACO^c^^,^^h^			
Yes	191	18.4 (15.0-21.8)	.02
No	115	12.4 (9.0-15.7)	

Abbreviation: ACO, accountable care organization.

^a^ Unweighted number of survey respondents.

^b^ Data from the OneKey database (IQVIA Inc).^20^

^c^ Data from the 2017-2018 National Survey of Healthcare Organizations and Systems.^19^

^d^ Data from the American Hospital Association’s Annual Survey were used to determine whether practices owned by a system also included a hospital with an academic medical center affiliation.^21^

^e^ Kaiser Family Foundation’s list of states that adopted Medicaid expansion at the time of survey administration was used for this information.^23^

^f^ Region was defined using US Census definitions.^24^

^g^ Rural was defined as an area with 49 999 or fewer residents using the rural urban commuting area definitions.^25^

^h^ The rate of missing data was equal to or greater than 10% for these variables.

### Characteristics of Hospitals Screening for Social Needs

We observed few significant differences among hospitals based on whether they screen for all needs: ownership, critical access status, delivery reform participation, rural status, region, and Medicaid expansion were all similar between groups. Academic medical centers were significantly more likely to screen patients for all needs compared with nonacademic medical centers (49.5%; 95% CI, 34.6%-64.4% vs 23.0%; 95% CI, 18.5%-27.5%; *P* < .001) (Table 2).

**Table 2.** Characteristics of Hospitals That Screen for Food Insecurity, Housing Instability, Utility Needs, Transportation Needs, and Experience With Interpersonal Violence

Characteristic	Screen for All 5 Needs		
	No.^a^	Weighted % (95% CI)	*P* Value
Overall	181	24.4 (20.0-28.7)	
Hospital ownership^b^			
Government	27	21.5 (13.3-29.7)	.79
Not for profit	123	25.4 (20.9-29.9)	
For profit	19	22.4 (1.7-43.0)	
Academic hospital^b^			
Yes	24	49.5 (34.6-64.4)	<.001
No	154	23.0 (18.5-27.5)	
Critical access hospital^c^			
Yes	36	19.7 (13.0-26.3)	.14
No	142	26.5 (20.7-32.3)	
Medicaid expansion state^d^			
Yes	112	26.7 (21.7-31.7)	.21
No	69	21.1 (14.2-28.1)	
Residents below federal poverty level^e^			
<11.5%	64	22.6 (16.0-29.1)	.70
11.5%-17.5%	50	23.9 (16.0-31.7)	
>17.5%	62	26.6 (19.5-33.8)	
Inpatient admissions with Medicaid as payer^b^			
<14.6%	56	21.2 (15.5-27.0)	.16
14.6%-21.1%	48	21.1 (12.2-30.0)	
>21.1%	65	30.3 (23.1-37.4)	
Region^f^			
South	68	25.2 (16.2-34.2)	.79
Midwest	51	21.4 (15.3-27.5)	
West	32	24.1 (15.9-32.4)	
Northeast	30	27.8 (16.9-38.8)	
Area^g^			
Rural	61	21.2 (15.9-26.4)	.15
Metropolitan	120	27.3 (20.3-34.2)	
Revenue from shared savings or capitation^c^			
Yes	90	24.6 (18.7-30.5)	.79
No	72	23.6 (17.9-29.4)	
Revenue from episode-based payments^c^			
Yes	88	24.8 (17.1-32.4)	.85
No	78	23.9 (18.5-29.2)	
Medicare ACO^b^^,^^h^			
Yes	66	26.9 (20.8-33.0)	.37
No	74	23.1 (17.2-29.0)	
Medicaid ACO^b^^,^^h^			
Yes	26	26.9 (17.3-36.4)	.62
No	114	24.2 (19.3-29.1)	
Commercial ACO^b^^,^^h^			
Yes	51	30.8 (23.0-38.7)	.09
No	89	22.7 (17.6-27.9)	

Abbreviation: ACO, accountable care organization.

^a^ Unweighted number of survey respondents.

^b^ Data from the American Hospital Association’s Annual Survey.^20^

^c^ Data from the 2017-2018 National Survey of Healthcare Organizations and Systems.^19^

^d^ Kaiser Family Foundation’s list of states that that adopted Medicaid expansion at the time of survey administration was used for this information.^23^

^e^ US Census Bureau data.^22^

^f^ Region was defined using US Census definitions.^24^

^g^ Rural was defined as an area with 49 999 or fewer residents using the rural urban commuting area definitions.^25^

^h^ The rate of missing data was equal to or greater than 10% for these variables.

We did not observe any differences in characteristics between organizations screening for some needs vs those screening for all 5 needs. When comparing organizations that screened for 0 needs, we observed some changes in significance. For physician practices, those that were not in an academic medical center were more likely to screen for 0 needs (34.5%; 95% CI, 31.0%-38.0% vs 27.6%, 95% CI, 23.3%-31.9%; *P* = .02); there were no significant differences by region, and there were no differences for commercial ACO contracts. For hospitals, nonacademic medical centers were not more likely to screen for 0 needs. There were significant differences such that critical access hospitals (15.1%; 95% CI, 8.7%-21.5% vs 4.9%, 95% CI, 2.9%-7.0%; *P* < .001), non–Medicaid expansion states (10.8%, 95% CI, 5.9%-15.8% vs 5.6%; 95% CI, 3.1%-8.1%; *P* = .05), rural hospitals (12.1%; 95% CI, 7.7%-16.5% vs 3.9%; 95% CI, 2.0%-5.7%; *P* < .001), hospitals without revenue from shared savings (11.0%; 95% CI, 6.7%-15.3% vs 4.4%; 95% CI, 2.4%-6.5%; *P* = .001), and hospitals without revenue from episode-based payments (10.9%; 95% CI, 6.7%-15.1% vs 4.4%; 95% CI, 1.9%-7.0%; *P* = .008) were all more likely to report screening for 0 needs.

### Barriers to Care Delivery Innovations

When asked about barriers to general dissemination of innovations, practices and hospitals that did not screen for social needs were significantly more likely to report a lack of financial resources, time, and incentives as major barriers (Table 3). For example, 51.0% (95% CI, 47.7%-54.4%) of practices that do not screen for social needs reported the lack of financial resources as a major barrier, whereas 37.5% (95% CI, 29.7%-45.9%) of practices that screen for all 5 needs reported this barrier (*P* = .001). Similarly, 60.0% (95% CI, 52.9%-66.8%) of hospitals that do not screen for social needs reported the lack of financial resources as a major barrier as opposed to 36.2% (95% CI, 28.6%-44.5%) of hospitals that screen for all 5 social needs (*P* < .001).

**Table 3.** Percentage of Physician Practices and Hospitals That Reported Barriers to Care Delivery Innovation, by Screening for Food Insecurity, Housing Instability, Utility Needs, Transportation Needs, and Experience With Interpersonal Violence

Barrier	Practices					Hospitals				
	No.^a^	Weighted % (95% CI)			*P* Value	No.^a^	Weighted % (95% CI)			*P* Value
		Not a Barrier	Minor Barrier	Major Barrier			Not a Barrier	Minor Barrier	Major Barrier	
Lack of financial resources										
Screens	334	23.8 (18.0-30.9)	38.7 (31.7-46.2)	37.5 (29.7-45.9)	.001	176	19.6 (13.4-27.7)	44.3 (35.5-53.4)	36.2 (28.6-44.5)	<.001
Does not screen	1757	14.1 (11.8-16.8)	34.8 (31.9-38.0)	51.0 (47.7-54.4)		523	10.4 (7.5-14.1)	29.6 (41.1-35.8)	60.0 (52.9-66.8)	
Lack of time										
Screens	336	24.2 (18.3-31.3)	47.3 (39.5-55.2)	28.5 (21.4-36.9)	<.001	176	33.7 (25.5-43.1)	48.2 (39.8-56.6)	18.1 (12.6-25.4)	.004
Does not screen	1761	13.2 (11.0-15.8)	36.1 (33.0-39.3)	50.7 (47.4-54.0)		522	21.7 (16.7-27.6)	43.6 (36.3-51.3)	34.7 (30.0-39.8)	
Lack of incentives										
Screens	333	32.1 (25.5-39.6)	42.9 (35.4-50.8)	25.0 (17.9-33.7)	.001	175	38.3 (29.9-47.3)	41.9 (34.1-50.2)	19.8 (13.7-27.8)	.003
Does not screen	1762	19.4 (17.0-22.0)	43.9 (40.6-47.2)	36.8 (33.6-40.1)		522	23.1 (18.5-28.5)	45.5 (40.7-50.3)	31.4 (26.4-36.9)	
Lack of knowledge										
Screens	333	34.0 (27.0-41.6)	49.3 (41.4-57.2)	16.8 (10.5-25.6)	.02	176	39.2 (30.4-48.7)	50.9 (41.9-59.8)	10.0 (5.6-17.0)	<.001
Does not screen	1763	22.3 (19.7-25.2)	54.9 (51.6-58.2)	22.7 (20.1-25.6)		523	22.8 (18.1-28.4)	50.1 (45.5-54.8)	27.1 (22.2-32.5)	
Lack of processes to identify innovations										
Screens	333	31.6 (24.9-39.1)	55.4 (47.5-63.0)	13.1 (8.9-18.8)	<.001	177	41.6 (33.1-50.6)	47.4 (38.7-56.2)	11.0 (6.5-18.0)	.008
Does not screen	1757	19.3 (16.9-22.0)	53.1 (49.8-56.4)	27.6 (24.5-30.8)		522	26.1 (20.9-31.1)	56.7 (49.1 -64.0)	17.2 (12.4-22.1)	
Lack of process for disseminating										
Screens	333	32.1 (25.4-39.6)	52.4 (44.4-60.2)	15.5 (10.8-21.9)	<.001	176	39.1 (30.4-48.5)	47.8 (38.9-56.9)	13.1 (8.0-20.9)	.01
Does not screen	1759	19.9 (17.4-22.6)	53.8 (50.5-57.1)	26.4 (23.6-29.4)		523	24.5 (19.8-30.1)	56.5 (51.9-61.1)	19.0 (14.2-24.9)	

^a^ Unweighted number of survey respondents from the 2017-2018 National Survey of Healthcare Organizations and Systems.

## Discussion

The link between social needs and health care outcomes has long been understood as important to achieving optimal health, yet this link has only recently been thrust into the spotlight as physicians and hospitals begin to take on accountability for population health and spending outcomes.^12,26,27,28,29^ These new data suggest that most US physician practices and hospitals are screening patients for at least 1 social need (most often, experience with interpersonal violence), and most are not screening patients for the 5 social needs that CMS has prioritized: food insecurity, housing instability, utility needs, transportation needs, and experience with interpersonal violence.^30^ Attention to the association of social needs with medical outcomes is widespread, but dissemination of care delivery innovations in physician practices and hospitals is notoriously slow^31^ and the barriers to linking medical and social care are high because funding streams and supportive organizations tend to be siloed.^32^ At the same time, despite the increasing recognition, we do not yet know if screening for patients’ social needs will translate into better care outcomes or lower spending because current evidence is limited.^33^ Given the lack of a clear evidence base coupled with steep barriers to addressing social needs within clinical care, it may be that physician practices and hospitals that are screening patients for social needs are early champions of screening.

Rates of screening vary by organizational characteristics, with hospitals and practices that serve more disadvantaged patients reporting higher screening rates. Nearly one-third of federally qualified health centers screen patients for all 5 needs. Practices with exposure to delivery and payment reform, including primary care improvement models, bundled payments, and commercial ACO contracts, are more likely to screen. Among hospitals, academic medical centers are more likely than nonacademic medical centers to screen patients for all social needs.

Hospitals and physician practices have different capabilities, needs, and motivations, which may influence the differential uptake of screening by hospitals. Physician practices may be motivated to screen patients for social needs to help provide more coordinated, comprehensive care in lower-cost settings (particularly if they participate in payment reform models and believe that addressing social needs will reduce health care spending), but they may lack the financial or staffing resources to routinely screen in the course of clinical care.^32^ Hospitals may have more resources, including staffing, financial, and technological, as well as more processes, protocols, and standardization in care delivery, which may explain higher screening rates among hospitals compared with physician practices.

Hospitals may also be more likely to screen patients for transportation and housing needs as part of their discharge processes because they are subject to federal regulations on patient safety as part of their certification from CMS.^34^ For example, given the patient’s medical condition, Medicare requires that hospitals assess whether a patient has adequate transportation from the hospital as well as a safe place to be discharged to.^35^ High-profile cases, such as the Maryland woman discharged from the emergency department on a street corner in the cold wearing a hospital gown, have garnered outcry from the public and the media.^36^ In response to such cases, some states have put in place stricter discharge planning requirements, especially in the emergency department given that patients with greater social needs and barriers to care are more likely to use the emergency department.^37,38^ For example, beginning in 2019, California emergency departments are required to have formalized discharge planning processes for homeless patients, to track discharges of homeless patients, and to provide these patients with clothing, food, and appropriate medication.^39^ Screening for social needs would be necessary to meet these requirements. Because patients with greater social needs are more likely to be readmitted,^40,41^ it is likely that hospitals have a financial motivation to screen for social needs.

Despite the spotlight on the importance of social needs, there is little consensus about responsibility for addressing social needs or the best approaches to the problem. For example, some health plans, particularly Medicaid managed care plans,^42^ are screening for social needs and implementing programs aimed at addressing patients’ needs. We hypothesize that other entities are likely screening for social needs as well, such as county social service agencies and schools. Although the health care system has an important role in addressing social needs, more discussion appears to be needed to minimize overlapping or duplicative work.

Physicians and hospitals, who are already strapped for time and have competing priorities, may be hesitant to screen patients for social needs when they have no real capacity or ability to address those needs,^43^ especially given the lack of robust studies behind screening.^33^ Physicians and hospitals likely have limited resources to help patients truly solve needs such as economic insecurity. Practices and hospitals in our study reported major barriers to innovation in clinical care, including a lack of resources, incentives, and time. Screening for social needs represents a major care-delivery innovation, and physicians and hospitals may need additional processes to link patients with local resources to address identified needs.^32^ Some payers directly support health care organizations in the work of implementing screening and referral processes. For example, North Carolina Medicaid’s Healthy Opportunities program is piloting a resource platform for physicians and hospitals.^14^ The National Association of Community Health Centers has developed a toolkit to aid physicians and hospitals in addressing social needs.^9^ UnitedHealthcare and the American Medical Association have partnered to produce new *International Statistical Classification of Diseases and Related Health Problems, Tenth Revision* codes so that physicians and hospitals can document patients’ social needs and payers can then refer patients to appropriate resources, which could reduce the burden on physicians and hospitals to manage treatment and assist patients with social needs.^44^

To implement screening protocols and begin addressing patients’ needs under a fee-for-service model, physicians and hospitals will need financial support. Payers could allow physicians and hospitals to bill for evidence-based programs, such as FoodRx, that have been shown effective at addressing needs and improving outcomes.^4^ The CMS could consider expanding care management billing to include managing care for patients who are both at risk or have clinically complex conditions in addition to social needs.

### Limitations

Our results should be interpreted in light of several limitations. This research is by nature descriptive, and our estimates are based on self-reported survey data from physician practices and hospitals. Senior leaders in hospitals and physician practices, who completed the survey, may not always know whether their organization screens patients for social needs. Furthermore, although our survey asks whether the organization has a “system in place” to screen patients for social needs, we do not know how systematically the organization is screening patients (eg, all patients vs those with specific payers vs those who have clinically complex conditions). Nor do we know how health care organizations are using patients’ screening results such as through offering referrals to community resources. Although weighted to represent the national landscape of practices and hospitals in our sample, there could be social desirability bias in our estimates, or leaders may not know about informal screening that takes place. Nevertheless, there is a paucity of systematic evidence regarding screening for and addressing social needs in mainstream clinical practice; we believe this study begins to fill this gap.^45^

Given the current focus on social needs from state and federal policymakers, payers, and physicians and hospitals, it seems likely that pressure on physicians and hospitals to identify and begin addressing patients’ social needs will continue. States are increasingly creating incentives for medical physicians, hospitals, and managed care organizations to integrate and address patients’ social needs.^29^ For example, Oregon’s Coordinated Care Organizations program will require participants to devote part of their budget to addressing social needs in 2020.^46^ The CMS has invested heavily in supporting social needs via their Accountable Health Communities project, which evaluates screening and referrals for social needs.^13^ Private payers are also active in this area: Blue Cross Blue Shield has food delivery programs, and UnitedHealthcare is piloting a centralized hub model for addressing social needs in key markets.^47,48^ Understanding which types of physician practices and hospitals are already screening patients for social needs can help policymakers know where to target their efforts. For example, nonacademic hospitals and physician practices that are not Federally Qualified Health Centers may need further incentives to screen patients.

## Conclusions

Although across stakeholders there is swelling momentum for addressing social needs, our study findings suggest that most physician practices and hospitals are not screening across 5 key social needs associated with health outcomes. We found that organizations participating in payment reform models were more likely to screen, and that organizations that do not screen reported misaligned incentives as a major barrier to innovations in care delivery. We believe systematic use of screening is a required first step to attend to social needs and improve health; addressing resource barriers, such as time, information, and money, may be a key element in supporting physicians and hospitals in efforts to screen patients for social needs.

## References

[1] Castrucci B, Auerbach J. Meeting individual social needs falls short of addressing social determinants of health. Health Affairs Blog. https://www.healthaffairs.org/do/10.1377/hblog20190115.234942/full/. Published January 16, 2019. Accessed August 13, 2019.

[2] Hood CM, Gennuso KP, Swain GR, Catlin BB County health rankings: relationships between determinant factors and health outcomes. Am J Prev Med. 2016;50(2):-. doi:10.1016/j.amepre.2015.08.024 26526164

[3] Solomon LS, Kanter MH Health care steps up to social determinants of health: current context. Perm J. 2018;22:18-139. doi:10.7812/TPP/18-139

[4] Goddu AP, Roberson TS, Raffel KE, Chin MH, Peek ME Food Rx: a community-university partnership to prescribe healthy eating on the South Side of Chicago. J Prev Interv Community. 2015;43(2):148-162. doi:10.1080/10852352.2014.973251 25898221 PMC4416784

[5] Hostetter M, Klein S, McCarthy D Hennepin Health: a care delivery paradigm for new Medicaid beneficiaries. The Commonwealth Fund website. https://www.commonwealthfund.org/publications/case-study/2016/oct/hennepin-health-care-delivery-paradigm-new-medicaid-beneficiaries. Published October 7, 2016. Accessed May 3, 2019.

[6] Kaufman A, Powell W, Alfero C, Health extension in new Mexico: an academic health center and the social determinants of disease. Ann Fam Med. 2010;8(1):73-81. doi:10.1370/afm.1077 20065282 PMC2807392

[7] Social determinants of health policy. American Academy of Family Physicians website. https://www.aafp.org/about/policies/all/social-determinants.html. Accessed April 5, 2019.

[8] Hagan JF, Shaw JS, Duncan PM, eds. Bright Futures: Guidelines for Health Supervision of Infants, Children, and Adolescents. 4th ed Elk Grove Village, IL: American Academy of Pediatrics; 2017.

[9] Protocol for Responding to Assessing Patients’ Assets, Risks, and Experiences (PREPARE). National Association of Community Health Centers website. http://www.nachc.org/research-and-data/prapare/. Updated 2019. Accessed April 9, 2019.

[10] Hamity C, Jackson A, Peralta L, Bellows J Perceptions and experience of patients, staff, and clinicians with social needs assessment. Perm J. 2018;22:18-105. doi:10.7812/TPP/18-105 30285914 PMC6172028

[11] Gottlieb L, Colvin JD, Fleegler E, Hessler D, Garg A, Adler N Evaluating the accountable health communities demonstration project. J Gen Intern Med. 2017;32(3):345-349. doi:10.1007/s11606-016-3920-y 27844261 PMC5331008

[12] Bachrach D Addressing patients' social seeds: an emerging business case for provider investment: report from the Commonwealth Fund; May 29, 2014 https://www.commonwealthfund.org/publications/fund-reports/2014/may/addressing-patients-social-needs-emerging-business-case-provider. Accessed August 13, 2019.

[13] Accountable health communities model. Centers for Medicaid & Medicare Services website. https://innovation.cms.gov/initiatives/ahcm/. Updated April 30, 2019. Accessed May 3, 2019.

[14] Healthy opportunities pilots fact sheet. North Carolina Department of Health and Human Services website. https://files.nc.gov/ncdhhs/SDOH-HealthyOpptys-FactSheet-FINAL-20181114.pdf. Published November 11, 2018. Accessed April 5, 2019.

[15] Artiga S, Hinton E Beyond health care: the role of social determinants in promoting health and health equity. 2018; https://www.kff.org/disparities-policy/issue-brief/beyond-health-care-the-role-of-social-determinants-in-promoting-health-and-health-equity/. Accessed April 5, 2019.

[16] LaForge K, Gold R, Cottrell E, How 6 organizations developed tools and processes for social determinants of health screening in primary care: an overview. J Ambul Care Manage. 2018;41(1):2-14. doi:10.1097/JAC.0000000000000221 28990990 PMC5705433

[17] Sundar KR Universal screening for social needs in a primary care clinic: a quality improvement approach using the your current life situation survey. Perm J. 2018;22:18-089. 30296397 10.7812/TPP/18-089 PMC6175598

[18] von Elm E, Altman DG, Egger M, Pocock SJ, Gøtzsche PC, Vandenbroucke JP; STROBE Initiative The Strengthening the Reporting of Observational Studies in Epidemiology (STROBE) statement: guidelines for reporting observational studies. Ann Intern Med. 2007;147(8):573-577. doi:10.7326/0003-4819-147-8-200710160-00010 17938396

[19] About NSHOS. Dartmouth Comparative Health System Performance website. https://sites.dartmouth.edu/coe/nshos/. Updated 2018. Accessed May 6, 2019.

[20] OneKey reference data set. IQVIA Inc. https://www.iqvia.com/locations/united-states/commercial-operations/essential-information/onekey-reference-assets. Accessed August 13, 2019.

[21] American Hospital Association. AHA Annual Survey Database. https://www.ahadata.com/aha-annual-survey-database-asdb/. Published 2017. Accessed August 13, 2019.

[22] US Census Bureau. American Community Survey (ACS). https://www.census.gov/programs-surveys/acs/about.html. Revised June 17, 2018. Accessed August 13, 2019.

[23] Status of state action on the Medicaid expansion decision. Kaiser Family Foundation website. https://www.kff.org/health-reform/state-indicator/state-activity-around-expanding-medicaid-under-the-affordable-care-act/?currentTimeframe=0. Updated May 13, 2019. Accessed May 13, 2019.

[24] US Census. Census Regions and Divisions of the United States. https://www2.census.gov/geo/pdfs/maps-data/maps/reference/us_regdiv.pdf. Published April 17, 2013. Accessed August 13, 2019.

[25] US Department of Agriculture. Rural-Urban Commuting Area Codes. https://www.ers.usda.gov/data-products/rural-urban-commuting-area-codes/. Updated July 3, 2019. Accessed August 13, 2019.

[26] Alley DE, Asomugha CN, Conway PH, Sanghavi DM Accountable health communities—addressing social needs through Medicare and Medicaid. N Engl J Med. 2016;374(1):8-11. doi:10.1056/NEJMp1512532 26731305

[27] Taylor LA, Tan AX, Coyle CE, Leveraging the social determinants of health: what works? PLoS One. 2016;11(8):e0160217. doi:10.1371/journal.pone.0160217 27532336 PMC4988629

[28] Ash AS, Mick EO, Ellis RP, Kiefe CI, Allison JJ, Clark MA Social determinants of health in managed care payment formulas. JAMA Intern Med. 2017;177(10):1424-1430. doi:10.1001/jamainternmed.2017.3317 28783811 PMC5710209

[29] Crumley D, Lloyd J, Pucciarello M, Stapelfeld B Addressing social determinants of health via Medicaid Managed Care contracts and Section 1115 Demonstrations. Center for Health Care Strategies. https://www.chcs.org/media/Addressing-SDOH-Medicaid-Contracts-1115-Demonstrations-121118.pdf. Published December 2018. Accessed April 5, 2019.

[30] Billioux A, Verlander K, Anthony S, Alley D Standardized screening for health-related social needs in clinical settings: the Accountable Health Communities screening tool. National Academy of Medicine. https://nam.edu/standardized-screening-for-health-related-social-needs-in-clinical-settings-the-accountable-health-communities-screening-tool/. Published May 30, 2017. Accessed August 13, 2019.

[31] Sidorov J Why health care innovation lags (and what to do about it). Health Affairs Blog. https://www.healthaffairs.org/do/10.1377/hblog20160803.056031/full/. Published August 3, 2016. Accessed April 5, 2019.

[32] Thomas-Henkel C, Schulam M Screening for social determinants of health in populations with complex needs: implementation considerations. Center for Health Care Strategies. https://www.chcs.org/media/SDOH-Complex-Care-Screening-Brief-102617.pdf. Published October 2017. Accessed April 5, 2019.

[33] Krista AH, Davidson K, Ngo-Metzger Q What evidence do we need before recommending routine screening for social determinants of health? Am Fam Physician. 2019;99(10):602-605. 31083876

[34] Centers for Medicare & Medicaid Services CMS manual system pub. 100-07 state operations provider certification 2008. https://www.cms.gov/Regulations-and-Guidance/Guidance/Transmittals/downloads/R37SOMA.pdf. Published October 17, 2008. Accessed March 11, 2019.

[35] Centers for Medicare & Medicaid Services Revision to state operations manual (SOM), hospital appendix A— interpretive guidelines for 42 CFR 482.43, discharge planning. https://www.cms.gov/Medicare/Provider-Enrollment-and-Certification/SurveyCertificationGenInfo/Downloads/Survey-and-Cert-Letter-13-32.pdf. Published May 17, 2013. Accessed April 5, 2019.

[36] McDaniels AK, Cohn M Woman found outside Baltimore hospital in gown and socks was experiencing psychotic episode, her lawyer says. *Baltimore Sun* https://www.baltimoresun.com/health/bs-hs-hospital-investigation-20180117-story.html. Published January 17, 2018. Accessed April 5, 2019.

[37] Capp R, Kelley L, Ellis P, Reasons for frequent emergency department use by Medicaid enrollees: a qualitative study. Acad Emerg Med. 2016;23(4):476-481. doi:10.1111/acem.12952 26932230

[38] Cheung PT, Wiler JL, Lowe RA, Ginde AA National study of barriers to timely primary care and emergency department utilization among Medicaid beneficiaries. Ann Emerg Med. 2012;60(1):4-10.e2. doi:10.1016/j.annemergmed.2012.01.035 22418570

[39] SB 1152, Hernandez. Hospital patient discharge process: homeless patients (Calif 2018). https://leginfo.legislature.ca.gov/faces/billTextClient.xhtml?bill_id=201720180SB1152. Published October 1, 2018. Accessed March 11, 2019.

[40] Meddings J, Reichert H, Smith SN, The impact of disability and social determinants of health on condition-specific readmissions beyond medicare risk adjustments: a cohort study. J Gen Intern Med. 2017;32(1):71-80. doi:10.1007/s11606-016-3869-x 27848189 PMC5215164

[41] Nagasako EM, Reidhead M, Waterman B, Dunagan WC Adding socioeconomic data to hospital readmissions calculations may produce more useful results. Health Aff (Millwood). 2014;33(5):786-791. doi:10.1377/hlthaff.2013.1148 24799575 PMC4079850

[42] Machledt D Addressing the social determinants of health through Medicaid managed care. https://www.commonwealthfund.org/publications/issue-briefs/2017/nov/addressing-social-determinants-health-through-medicaid-managed. Published November 29, 2017. Accessed April 5, 2019. 29235781

[43] Olayiwola JN, Willard-Grace R, Dubé K, Higher perceived clinic capacity to address patients’ social needs associated with lower burnout in primary care providers. J Health Care Poor Underserved. 2018;29(1):415-429. doi:10.1353/hpu.2018.0028 29503309

[44] UnitedHealthcare and the AMA collaborate to understand and address social barriers preventing people’s access to better health [news release]. https://www.unitedhealthgroup.com/newsroom/2019/2019-04-02-uhc-ama-social-barriers.html. Published April 2, 2019. Accessed April 17, 2019.

[45] Fraze T, Lewis VA, Rodriguez HP, Fisher ES Housing, transportation, and food: how ACOs seek to improve population health by addressing nonmedical needs of patients. Health Aff (Millwood). 2016;35(11):2109-2115. doi:10.1377/hlthaff.2016.0727 27834253 PMC5377443

[46] Clary A Oregon’s accountable health model addresses health equity and health-related needs: four lessons from CCO 2.0. State Health Policy Blog. https://nashp.org/oregons-accountable-health-model-addresses-health-equity-and-health-related-needs-four-lessons-from-cco-2-0/. Published March 11, 2019. Accessed March 11, 2019.

[47] Shropshire C Blue Cross launches healthy food delivery service to Chicago food deserts. *Chicago Tribune* https://www.chicagotribune.com/business/ct-biz-food-delivery-service-food-desert-20190212-story.html. Published February 12, 2019. Accessed April 5, 2019.

[48] Goldman TR Charting a pathway to better health. Health Aff (Millwood). 2018;37(12):1918-1922. doi:10.1377/hlthaff.2018.05166 30633689

